# Resistance Modulation of Individual and Polymicrobial Culture of *S. aureus* and *E. coli* through Nanoparticle-Coupled Antibiotics

**DOI:** 10.3390/biomedicines11112988

**Published:** 2023-11-07

**Authors:** Sana Zia, Song Peng, Arslan Bashir, Tasleem Kausar, Shanza Rauf Khan, Afshan Muneer, Attia Nawaz, Lina I. Alnajjar, Mohd Saeed, Nawaf Alshammari, Amjad Islam Aqib, Kun Li

**Affiliations:** 1 MOE Joint International Research Laboratory of Animal Health and Food Safety, Institute of Traditional Chinese Veterinary Medicine, College of Veterinary Medicine, Nanjing Agricultural University, Nanjing 210095, China; sanazia389@gmail.com (S.Z.); payson@stu.njau.edu.cn (S.P.); 2 Department of Zoology, The Government Sadiq College Women University, Bahawalpur 61300, Pakistan; tasleem.kausar@gscwu.edu.pk; 3 Department of Chemistry, University of Agriculture, Faisalabad 38000, Pakistanshanzaraufkhan@gmail.com (S.R.K.); 4 Department of Zoology, Cholistan University of Veterinary and Animal Sciences, Bahawalpur 63100, Pakistan; afshanchudhary9@gmail.com; 5 Department of Microbiology, Cholistan University of Veterinary and Animal Sciences, Bahawalpur 63100, Pakistan; attianawaz555@gmail.com; 6 Department of Pharmacy Practice, College of Pharmacy, Princess Nourah bint Abdulrahman University, Riyadh 11671, Saudi Arabia; lialnajjar@pnu.edu.sa; 7 Department of Biology, College of Science, University of Hail, P.O. Box 2440, Hail 34464, Saudi Arabia; mo.saeed@uoh.edu.sa (M.S.); naib.alshammari@uoh.edu.sa (N.A.); 8 Department of Medicine, Cholistan University of Veterinary and Animal Sciences, Bahawalpur 63100, Pakistan

**Keywords:** mixed culture, *E. coli*, *S. aureus*, tungsten oxide nanoparticles, antibiotics, antibiotics-coupled nanoparticles

## Abstract

Polymicrobial mastitis is now becoming very common in dairy animals, resulting in exaggerated resistance to multiple antibiotics. The current study was executed to find drug responses in individual and mixed Culture of *Staphylococcus aureus* and *Escherichia coli* isolated from milk samples, as well as to evaluate the antibacterial potential of tungsten oxide nanoparticles. These isolates (alone and in mixed culture) were further processed for their responses to antibiotics using the disc diffusion method. On the other hand, tungsten oxide WO_3_ (W) nanoparticles coupled with antibiotics (ampicillin, A, and oxytetracycline, O) were prepared through the chemical method and characterized by X-ray diffraction, scanning electron microscopy (SEM), and UV-visible techniques. The preparations consisting of nanoparticles alone (W) and coupled with ampicillin (WA) and oxytetracycline (WO) were tested against individual and mixed Culture through the well diffusion and broth microdilution methods. The findings of the current study showed the highest resistance in *E. coli* was against penicillin (60%) and ampicillin (50%), while amikacin, erythromycin, ciprofloxacin, and oxytetracycline were the most effective antibiotics. *S. aureus* showed the highest resistance against penicillin (50%), oxytetracycline (40%), and ciprofloxacin (40%), while, except for ampicillin, the sensitive strains of *S. aureus* were in the range of 40–60% against the rest of antibiotics. The highest zones of inhibition (ZOI) against mixed Culture were shown by imipenem and ampicillin, whereas the highest percentage decrease in ZOI was noted in cases of ciprofloxacin (−240%) and gentamicin (−119.4%) in comparison to individual Culture of *S. aureus* and *E. coli*. It was noteworthy that the increase in ZOI was not more than 38% against mixed Culture as compared to the individual Culture. On the other hand, there was a significant reduction in the minimum inhibitory concentration (MIC) of nanoparticle-coupled antibiotics compared to nanoparticles alone for individual and mixed-culture bacteria, while MICs in the case of mixed Culture remained consistently high throughout the trial. This study therefore concluded that diverse drug resistance was present in both individual and mixed-culture bacteria, whereas the application of tungsten oxide nanoparticle-coupled antibiotics proved to be an effective candidate in reversing the drug resistance in bacterial strains.

## 1. Introduction

Developing countries like Pakistan experience two-pronged challenges in the form of horizontal expansion of dairy animals and prevailing dairy udder challenges, overall jeopardizing health, and the economy. Such scenarios lead to a daily shortfall of milk for the consumers, e.g., Karachi, a cosmopolitan city of Pakistan, has reached an uncertain supply of up to 4 million liters per day. In addition, it is expected that milk consumption will find a minimum annual growth rate of 5% soon [1]. The dairy sector is currently facing a serious threat in the form of mastitis, which leads to a notable decrease in the yield and quality of milk, along with a sharp increase in treatment expenses and bovine mortality [2]. Furthermore, the augmentation of milk production is hampered by bacterial attacks in the udders of dairy animals, putting both animal and public health at risk [3]. Dairy cows are susceptible to more than 150 types of bacteria that can cause mastitis, including *S. aureus*, *Mycoplasma* species, *Streptococcus uberis*, *Streptococcus dysgalactiae*, *Escherichia coli*, and *Klebsiella pneumoniae* [4], which are responsible for the development of a wide variety of lesions [5]. In veterinary practice, the use of antibiotics often goes unjustified, culminating in development of microbial resistance and hence compromising animal and public health [6]. Microorganisms evolve antimicrobial resistance to survive in continuously changing environments [7]. Recent investigations have documented substantial fluctuations in the efficacy of antibiotics against pathogens such as *E. coli* and *S. aureus*, providing clear indications of their escalating resistance tendencies [8,9].

The wise approach in the current scenario is the five R concept, specifically, responsibility, refinement, reduction, replacement, and review of antimicrobial use (AMU). To reduce the burden of antibiotics or to replace antibiotics, alternatives are needed. As an alternative, nanoparticles are thought to work differently than antibiotics and can help reduce drug resistance by serving as carriers for antibiotics, having synergistic effects with antimicrobials and being antimicrobials themselves. Nanoparticles larger than 10 nm have been found to cause cytotoxicity and cellular disintegration through their interaction with the cellular wall and membrane constituents [9]. The integration of antibacterial agents into biomaterials is a strategic approach aimed at mitigating these challenges. In medicine and pharmaceuticals, nanotechnology plays a crucial role [10]. Apart from their higher reactivity, these particles are unique in their physicochemical properties, which include lower surface-to-volume ratios, greater stability, bioactivity, and bioavailability [11]. Recent studies showed that combining nanoparticles with antibiotics, antimicrobial peptides, and essential oils minimizes the potentially toxic effects of nanoparticles [12]. Tungsten oxide coating has found novel applications in antibacterial coatings, where it helps to improve the antibacterial efficacy of surgical equipment and medical devices [13]. Researchers discovered that WO_3_-X nanodots exhibited strong bactericidal activity after they were exposed to membrane stress [14]. WO_3_-X nanodots demonstrate a remarkable capability to eliminate both *Escherichia coli* and *Staphylococcus aureus* [15]. Furthermore, previous studies have demonstrated that WO_3_ showed promising antimicrobial activity against many bacterial strains, including *E. coli*, *P. multocida*, *B. subtilis* (gram-positive), and *S. aureus* (gram-positive) [16].

The study of nanoparticle coupling with antibiotics targeting *E. coli* and *S. aureus* is of significance because it can helps combat antimicrobial resistance, boost the antimicrobial activity of these drugs, facilitate targeted drug delivery, support combination therapies, advances the diagnosis of various bacterial-related diseases, and expedite the healing process. Hence, the purpose of this research article was to investigate responses of individual and mixed culture *Staphylococcus aureus* and *E. coli* against antibiotics, and to evaluate tungsten oxide nanoparticle-coupled antibiotics as drug resistance modulators.

## 2. Materials and Methods

### 2.1. Sample Collection

The sample collection area encompassed districts within the Bahawalpur region, chosen based on accessibility and the consent of dairy farmers. Milk samples were aseptically collected in sterile vials using the convenience statistical method to reach a total sample number of *n* = 200 [17]. The samples were screened at the time of collection for subclinical mastitis following the protocol described by Muhammad et al. [18]. The positive samples were delivered to the laboratory of the Department of Microbiology at Cholistan University of Bahawalpur in a container maintained at a temperature of 4 °C.

### 2.2. Isolation of E. coli and S. aureus

The milk samples were put into incubation for 24 h at 37 °C, following which centrifugation at a speed of 3634× *g* for 5 min was conducted. The characteristic bacterial colonies were streaked on differential media of both bacteria, i.e., mannitol salt agar for *S. aureus* and MacConkey agar for *E. coli*. The plates were incubated at 37 °C for another 24 h. The mannitol salt agar was transformed into a yellow color with pinpoint round colonies on the media for *S. aureus*, while MacConkey agar was transformed into pink colonies on the media for *E. coli*. These colonies were further subjected to a series of biochemical assays as described by Bergey’s manual of determinative bacteriology [19]. The confirmation of *S. aureus* and *E. coli* was determined using pooled information obtained from microbiological and biochemical experiments.

### 2.3. Antibiotic Susceptibility of E. coli and S. aureus

To determine the susceptibility of *E. coli*, *S. aureus*, and mixed Culture of *E. coli* and *S. aureus*, a total of eight antibiotics (erythromycin, ciprofloxacin, imipenem, amikacin, ampicillin, oxytetracycline, and gentamicin) were tested. The selection of these antibiotics was based on their common usage in clinical laboratories and adherence to the guidelines provided by the Clinical Laboratory and Standard Institute. Some of the antibiotics had previously been used in other studies (unpublished data or under review for publication) of the authors so have been exempted from this study. The Kirby–Bauer disc diffusion method was used to determine zones of inhibition, which were then compared with the standards provided by the Clinical Laboratory and Standard Institute [20]. In brief, fresh *E. coli* and *S. aureus* growth was adjusted at 1–1.5 × 10^8^ CFU/mL (colony-forming units/milliliter) on sterile Mueller–Hinton agar and antibiotic discs were put aseptically at equal distances from one another. The agar plates were incubated at 37 °C for 20–24 h, and zones were measured with vernier calipers and compared with the standard zones to identify resistant, intermediate, and sensitive strains of *S. aureus* and *E. coli* [20]. *S. aureus* subspecies *aureus* ATCC 25923^TM^ and *E. coli* ATCC25922 were used as a control strain in this study.

### 2.4. Response of Mixed Culture against Different Antibiotics

The disc diffusion method was employed to assess the susceptibility of mixed Culture consisting of *S. aureus* and *E. coli* against various antibiotics. For this purpose, a total of three samples each of *S. aureus* (S) and *E. coli* (E) were randomly selected from the previous trial (Section 2.3). These samples were mixed with each other to create three mixed culture samples, namely S1E1, S2E2, and S3E3. Briefly, the 0.5 McFarland solution of each bacterial strain was combined in equal proportion to make a resulting concentration of 0.5 McFarland (equal to 1–1.5 × 10^8^ CFU/mL) for the SE combination. This study involved the examination of both individual bacteria and mixed Culture to assess their responses to a total of eight antibiotics with the same protocol as mentioned in Section 2.3 of this manuscript. Zones of inhibition (ZOI) were measured and compared, and comparisons of each individual and mixed culture were made. The percentage increase or decrease in ZOI values of mixed Culture with individual bacteria was calculated to evaluate the extent of variation exhibited by mixed Culture against antibiotics.

### 2.5. Synthesis and Characterization of Nanoparticle-Coupled Antibiotics

#### Synthesis of WO_3_ Nanoparticles

Sodium tungstate (Na_2_WO_4_) and cetyltrimethylammonium bromide (CTAB) were individually dissolved in deionized water using a solution of 6 g of Na_2_WO_4_ and 18 mL of deionized water. Both solutions were mixed, and a small amount of hydrochloric acid (HCl) was added to adjust the pH to within the range of 1–2. This reaction mixture was autoclaved at 80 °C in a 100 mL Teflon vessel for 4 days in a stainless-steel lined autoclave. The hydrothermally precipitated materials were filtered and rinsed with deionized water and ethanol at regular intervals after hydrothermal treatment. After precipitation, the precipitates dried at 120 °C for two hours. A further 3.5 h of calcination at 500 °C was completed. After calcination, the product was ground into powder with a pestle and mortar.

Ampicillin and oxytetracycline were selected for this trial based on their clinical use, resistance profile, and better coupling ability with nanoparticles in a pilot study. These antibiotics were dissolved in 20 mL of deionized water, while 1.5 g of nanoparticles were dispersed in deionized water and stirred for five hours at room temperature in the presence of PVP dissolved in 20 mL of deionized water. The solutions were stored at room temperature for 24 h with regular mixing and stirring. The product was centrifuged for 30 min at 3000 rpm to settle at the bottom. A mortar and pestle were used to grind the product after it was dried for 8 h at 100 °C. The absorbance of coated nanoparticles was measured using a US-1100 double beam UV-visible spectrophotometer from LYNX, LM-56-1001AE in Pakistan. We performed XRD using a Rigaku TTR instrument (Tokyo, Japan) at 40 kV and 300 mA, in the range 2θ between 20 and 80, with Cu kα (λ) radiation around 0.15406 nm, to investigate the crystal structure of nanoparticles. An FTIR analysis was conducted using an FTIR spectrometer (Perkin Elmer Spectrum America) at room temperature within the spectral range of 4000–500 cm^−1^. Scanning images and elemental analysis were performed on a TESCAN MIRA 3 (Brno, Czech Republic), which is available at the Institute of Space Technology, Islamabad, Pakistan. The Raman spectra were collected using a Peak Seeker Pro-Agiltron Raman spectrometer (USA) with a laser light source of 50 mW at 785 nm. At room temperature, 50 mg of each sample were placed on aluminum slides for examination. Thermal analysis was executed in a controlled nitrogen environment employing a Q600 SDT thermogravimetric analyzer with a heating rate of 10 °C per minute. The analysis of sample mass changes was performed using a POWEREACH JC 2000D2W contact angle tester produced in Pakistan.

### 2.6. Resistance Modulation by Nanoparitcle Coupled Antibiotics against Individuals and Mixed Culture

An empirical technique was used to estimate the antibacterial activity of WO_3_-coated antibiotics. The minimum inhibitory concentrations (MICs) of the preparations were determined using broth microdilutions.

#### 2.6.1. Agar Well Diffusion Method

A fresh culture of individual *E. coli* and individual *S. aureus* was adjusted to 1–1.5 × 10^8^ CFU/mL by obtaining turbidity of culture equal to 0.5 McFarland. To make the final adjustment at 1–1.5 × 10^8^ CFU/mL, a 1/2:1/2 ratio of both bacteria (each having 0.5 McFarland solutions equaling 1–1.5 × 10^8^ CFU/mL) was used to generate the mixed culture for the agar well diffusion method. This was achieved by combining half of an *E. coli* solution (1–1.5 × 10^8^ CFU/mL) and half of a *S. aureus* (1–1.5 × 10^8^ CFU/mL) solution to make a total of 1 mL (0.5 McFarland). The culture was spread on Mueller–Hinton agar homogenously. The well borer was used to make wells (6–8 mm) on sterile Mueller–Hinton agar at equal distances. Tungsten oxide nanoparticles alone and tungsten oxide nanoparticles coupled with antibiotics were poured into the wells (15 μL of 0.01 gm/mL) and incubated at 37 °C for 24 h. The zones of inhibition (ZOI) produced by this preparation against *E. coli*, *S. aureus*, and mixed culture were measured using vernier calipers [21].

#### 2.6.2. Minimum Inhibitory Concentration (MIC)

Sterile broth was poured in all wells of sterile 96-well titration plate. Two-fold serial dilutions, starting from 10,000 µg/mL of each preparation were carried out until the 11th well. A fresh growth of *E. coli* and *S. aureus* adjusted at 1 × 10^5^ CFU/mL was poured in all wells except the negative control. To make the final adjustment at 1 × 10^5^ CFU/mL for mixed Culture, the solutions of *E. coli* (1 × 10^5^ CFU/mL) and *S. aureus* (1 × 10^5^ CFU/mL) were combined in a 1/2:1/2 ratio. Positive control (well containing both broth and culture) and negative control (well containing only broth) were reserved in the 12th column. The optical density at 600 nm wavelength was measured after 0, 4, 20, 24, and 28 h of incubation. In this study, OD values were taken at the 4 h duration both before and after the standard time of incubation, i.e., 24 h. This was intended to find the first minimum dosage if the infection is to be tackled on an immediate basis, as well as the hours around the infection period.

A net OD value was determined by comparing the values taken with those obtained after 0 h of incubation. Various concentrations were tested for inhibition of growth based on the net OD value. The minimum inhibitory concentration (MIC) was determined to be the lowest concentration of preparation that inhibited the growth of bacteria [22].

### 2.7. Statistical Analysis

Parametric tests, *t*-tests, and analysis of variance (ANOVA) were applied to the data obtained from disc diffusion, well diffusion, and the broth microdilution method. Tukey’s test was applied in conjunction with ANOVA to compare the means for statistically significant differences. SPSS version 22 was used to analyze the data at *p* < 0.05.

Formulae Used

$$\%\mathrm{Change}\;\mathrm{in}\;\mathrm{ZOI}\;\mathrm{of}\;\mathrm{mixed}\;\mathrm{culture}\;\mathrm{compared}\;\mathrm{with}\;\mathrm{the}\;\mathrm{individual}\;\mathrm{culture}\;\mathrm{bacteria}\;\;=\frac{\left(\mathrm{ZOI}\;\mathrm{of}\;\mathrm{mixed}\;\mathrm{culture}\;\mathrm{bacteria}-\mathrm{ZOI}\;\mathrm{of}\;\mathrm{individual}\;\mathrm{culture}\;\mathrm{bacteria}\right)}{\mathrm{ZOI}\;\mathrm{of}\;\mathrm{mixed}\;\mathrm{culture}\;\mathrm{bacteria}\;}\times 100$$

## 3. Results

### 3.1. Characterization of Nanoparticles

#### 3.1.1. Tungsten Oxide

**Figure 1:**
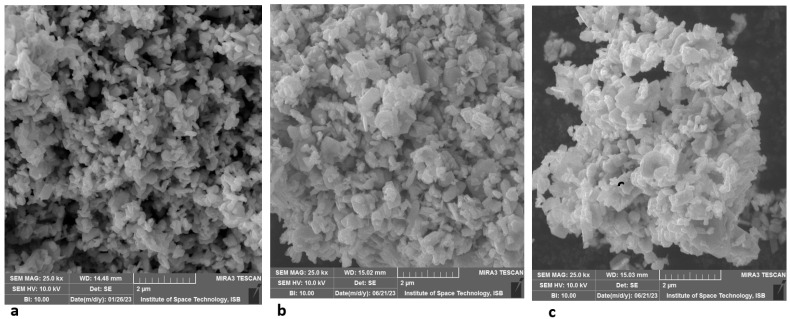
Scanning electron microscopic images of tungsten oxide nanoparticles alone and coated with antibiotics. (**a**) SEM image of tungsten oxide nanoparticle, (**b**) SEM image of tungsten oxide nanoparticles coupled with ampicillin, (**c**) SEM image of tungsten oxide nanoparticles coupled with oxytetracycline.

The FTIR spectra of tungsten oxide (WO_3_) within the range of 1000 to 500 cm^−1^ refer to the characteristic lattice vibration exhibited by tungsten oxide nanoparticles. The stretching vibration of W-O-W and the bending vibration of W-O and W=O are characterized by a prominent peak at around 799 cm^−1^. The peak at 1600 cm^−1^ is described as a vibration of the symmetrical OH of the hydroxyl group as well as a W-OH phase interaction. The utilization of an acidic medium during the preparation of the sample may yield larger crystallites with higher concentrations of oxygen defects than the sample prepared in less acidic media. Consequently, the symmetrical vibrations of the W-OH, H_2_O, and W-OH molecules were affected. A strong stretching W-O-W response in the inorganic compound itself is visible at 799 cm^−1^. The correlation between vibration and sample preparation was observed to be stronger when using more acidic media, such as a pH value of 2. Figure 1 represents a comprehensive view of the product, where the WO_3_ nanoparticles exhibit an array of randomly arranged particles. Upon closer examination, the nanoparticle surface has a smooth texture, characterized by spheroidal or oval morphology. The nanoparticles of WO_3_ are oval with rounded ends and their respective ends intersect at different points. Figure 1a displays the scanning electron microscopy (SEM) images depicting the synthesized product WO_3_, which was obtained using hydrothermal methods. It was observed that a drug coating had been applied to the WO_3_ nanoparticles, resulting in a variation in absorbance with wavelength. However, the absorbance pattern exhibited several peaks.

#### 3.1.2. Tungsten Oxide Coupled Antibiotics

**Figure 2:**
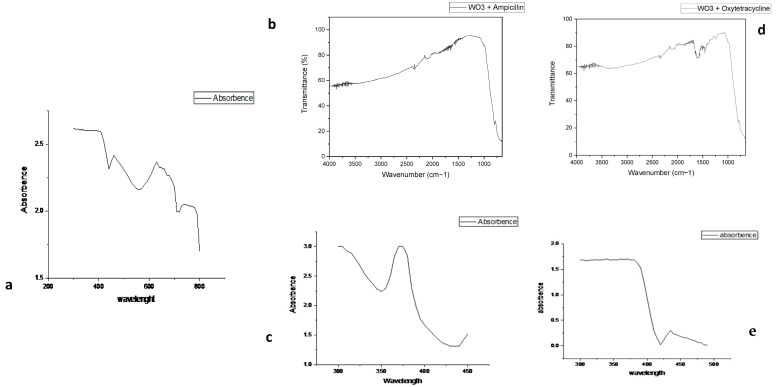
FTIR spectra and UV-visible spectra of tungsten oxide nanoparticles and antibiotics. (**a**) UV-visible spectra of tungsten oxide nanoparticle, (**b**) FTIR spectra of WO_3_ + ampicillin, (**c**) UV-visible spectra of oxytetracycline, (**d**) FTIR spectra of WO_3_ + ampicillin, (**e**) UV-visible spectra of ampicillin.

It has been observed in previous studies that the most characteristic bands of oxytetracycline (OTC) fall in the 1100 cm^−1^ to 1700 cm^−1^ region. Peaks at 1653 cm^−1^ to 1522 cm^−1^ were assigned to the -C=O and -NH_2_ groups of the amide group in ring A, respectively. The peaks at 1617 cm^−1^ and 1541 cm^−1^ were attributed to the -C=O group in ring C and amide -NH, respectively. The 1457 cm^−1^ peak, on the other hand, was attributed to C=C skeletal vibration. The O-H stretching vibration in the alcohol and phenolic groups is responsible for the intense stretching bond centered between 3300 cm^−1^ to 3500 cm^−1^. When a tungsten oxide nanoparticle (WO_3_) is associated with it, a tungsten oxide appears at 799 cm^−1^, indicating that the W-O-W stretching vibration is present. These peaks confirmed the presence of pure WO_3_ nanoparticles in a sample and their interaction with OTC (Figure 2).

The peak at 799 cm^−1^ indicates that W-O-W stretching vibrations are present in WO_3_. The prominent peak at 1550 cm^−1^ indicates that the C-C stretching vibration of the aromatic ring is present in the sample. The presence of an alkene =C-H bend is shown by the peak at 1004 cm^−1^. The peak from 1680 cm^−1^ to 1630 cm^−1^ reveals the presence of a C=O stretching vibration in the amide group, together with the presence of two alkyl groups. Additionally, the bending vibrations of CH_3_ and CH_2_ can be observed at 1475 cm^−1^ and 1365 cm^−1^, respectively. The C=O stretching vibration in the carboxylic group generally exhibits a peak within the range of 1760 cm^−1^ to 1665 cm^−1^ in. The peak at 1585 cm^−1^ corresponds to the vibrational mode associated with the stretching of C-C bonds within the aromatic ring. The peak at 799 cm^−1^ confirms the presence of pure WO_3_ nanoparticles in the sample and its interaction with ampicillin (Figure 2).

### 3.2. Antibiotic Susceptibility of Individual Bacteria

The response of *E. coli* against different antibiotics was more inclined towards the resistant category when compared to *S. aureus* (Table 1). None of the *E. coli* isolates showed less than 20% resistance, however the highest percentage of resistant *E. coli* being 60% against penicillin. In contrast, it was observed that none of the isolates exhibited a sensitivity of less than 30% against any antibiotics tested in this study. The highest percentage of sensitive strains (60%) were noted against imipenem, amikacin, and erythromycin. Subsequently, the percentages of sensitive isolates were noted to be 50% for gentamicin, 40% for erythromycin, penicillin, and ciprofloxacin, and 30% for ampicillin. The largest percentage of intermediate isolates was higher in the case of *S. aureus* as compared to those of *E. coli*. The highest proportion of intermediate susceptible isolates was reported in 40% of *S. aureus* cases against ampicillin, followed by 30% against imipenem, while in *E. coli* cases, the percentage of intermediate susceptible isolates remained between 10 and 20% against different antibiotics.

### 3.3. Response of Mixed-Culture Bacteria against Antibiotics

**Figure 3:**
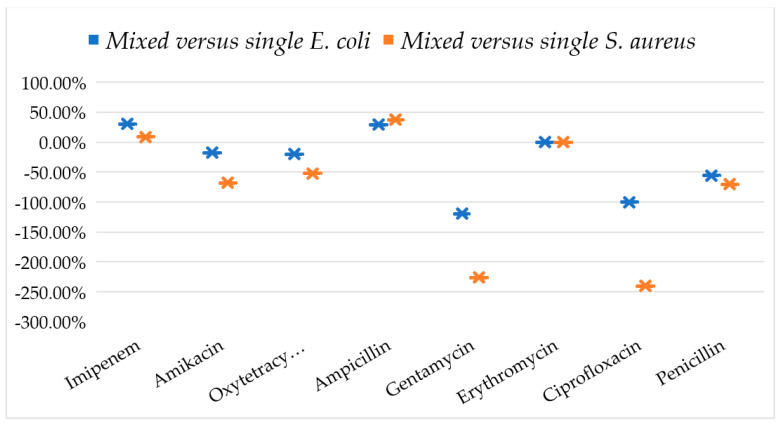
Percentage variation (increase/decrease) in zones of inhibition of mixed culture (*E. coli* plus *S. aureus*) in comparison to individual-culture *E. coli* and individual culture *S. aureus* against different antibiotics. This percentage was measured as “(ZOI of mixed culture − ZOI of individual culture)/mixed culture × 100”.

A significant difference in the ZOI was observed among individual and mixed-culture bacteria when exposed to various antibiotics. The findings of this study challenge the commonly held belief that the reduction in ZOI is a consistent outcome in mixed Culture. Specifically, the results indicate that certain combinations of antibiotics, such as imipenem and ampicillin, exhibited increased susceptibility in terms of higher zones of inhibition compared to individual culture bacteria (Table 1). The variability in the response of mixed-culture bacteria in comparison to individual culture bacteria to different antibiotics was observed, with instances of both elevated and dramatically lowered responses (Figure 3). This study revealed that the mixed culture exhibited 37.67% and 8.69% higher ZOI compared to the individual *S. aureus* when exposed to ampicillin and imipenem, respectively. However, when tested against other antibiotics, there was a significant decrease in ZOI, except for erythromycin, where no change in ZOI was observed. The most significant reduction in ZOI was observed while testing ciprofloxacin (−240%) followed by gentamicin (−225.94%), penicillin (−70.60%), amikacin (−67.84%), and oxytetracycline (−52.10%) in comparison with individual *S. aureus*. Similarly, the mixed-culture bacteria in comparison to individual *E. coli* showed an increase in percentage ZOI only in the cases of imipenem (30.4%) and ampicillin (29.51%). Gentamicin caused the highest percentage decrease in ZOI in mixed culture over individual *E. coli* (−119.4%), followed by ciprofloxacin (−100%), penicillin (−55.95%), oxytetracycline (−20.04%), and amikacin (−17.89%). Mixed Culture versus individual *E. coli* showed lesser percentages of decreasing in ZOI when compared to the mixed Culture versus individual *S. aureus*. The findings of this study indicate that the *E. coli* bacteria exhibited a higher level of resistance compared to *S. aureus.* Furthermore, the presence of *E. coli* in polymicrobial (mixed) Culture proved to be the major factor contributing to the observed resistance.

### 3.4. Antibacterial Potential of Nanoparticle-Coupled Antibiotics against individual and Mixed-Culture Bacteria

**Figure 4:**
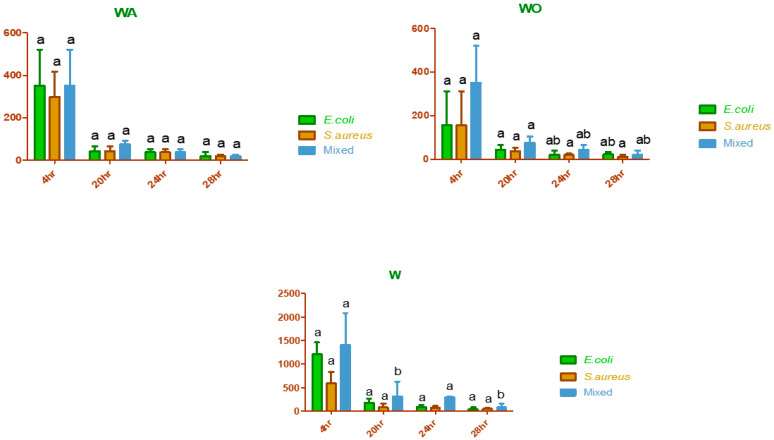
Comparison of responses among individual *E. coli*, individual *S. aureus*, and mixed-culture bacteria against nanoparticles alone and nanoparticle-coupled antibiotics at different incubation periods. Y-axis shows minimum inhibitory concentrations (µg/mL) of *E. coli*, *S. aureus*, and mixed Culture while x-axis shows incubation periods (hours). Different superscripts against each preparation at each incubation interval indicate significant differences (*p* < 0.05), WA = Tungsten oxide nanoparticle coupled ampicillin, WO = Tungsten oxide nanoparticle coupled oxytetracycline, W = Tungsten oxide nanoparticle alone.

**Figure 5:**
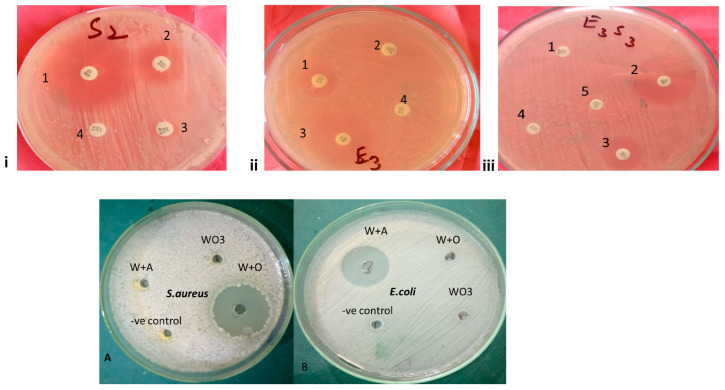
Antibacterial potential of antibiotics through disc diffusion (**i**–**iii**) and nanoparticle-coupled antibiotics (**A**,**B**) through well diffusion method. 1,2,3,4,5 are indicating different antibiotics, Handwritten S_2_ and E_3_ are individual bacteria of *S. aureus* and *E. coli*, respectively while E_3_S_3_ mixed culture, (**i**) = Antibiotic susceptibility of *S. aureus*, (**ii**) = Antibiotic susceptibility of *E. coli*, (**iii**) = Antibiotic susceptibility of mixed culture of *S. aureus* and *E. coli.* Plates A and B show zones of inhibition produced against *S. aureus* (**A**) and *E. coli* (**B**), respectively, by oxide nanoparticles (WO_3_), tungsten oxide, tungsten oxide coupled with ampicillin (W + A), tungsten oxide, and tungsten oxide coupled with oxytetracycline (W + O).

The MICs of tungsten oxide (W) nanoparticles alone and tungsten oxide nanoparticle-coupled oxytetracycline (WO) and ampicillin (WA) against specific *E. coli* showed significant differences (*p* < 0.05). A similar response was noticed in the cases of individual *S. aureus* and mixed Culture. The highest MIC (2083.33 ± 721.69 µg/mL) was noted in the case of W against mixed culture after 4 h of incubation, while the lowest (19.53 ± 0.00 µg/mL) was noted in the case of WO against individual *S. aureus* after 28 h of incubation (Table 2). However, it was noteworthy that a non-significant difference (*p* < 0.05) in MICs was found between WA and WO at all types of tested incubation periods. The results indicate that the MIC for W in mixed-culture bacteria was found to be higher in comparison to that of individual bacteria (Figure 4 and Figure 5). The MIC of WA against mixed culture, when compared to *E. coli* alone, exhibited elevated values across all types of tested incubation periods except at the 20th and 28th hours of incubation. During this period, no changes in MIC or reduction in MIC, respectively, were observed for the mixed culture in comparison to the individual *E. coli*. Except for the 24th and 28th hours of incubation (where the MIC remained unchanged), there was an observed increase in the MIC of the mixed culture in comparison to that of individual *S. aureus*. With the response of mixed Culture in comparison to those of individual *S. aureus* and individual *E. coli*, it was shown that the application of WO resulted in an increase in MIC at different incubation periods.

There was a statistically significant decrease *(p* < 0.05) in the MIC after 4 h of incubation for each of W, WA, and WO against *S. aureus*, *E. coli*, and mixed-culture bacteria. However, the reduction in MIC remained stable after 24 h without any significant change. The data also indicated that maximum effectiveness in terms of reduced MIC may be attained following a 20 h incubation period. This trend was found to be equally applicable for W, WA, and WO against *S. aureus* alone, *E. coli* alone, and mixed culture (Table S1, Figure 3).

## 4. Discussion

### 4.1. Characterization of Nanoparticle-Coupled Antibiotics

There is a clear peak in our current FTIIR spectra around 799 cm^−1^, which is a signal of stretching vibrations in W-O-W and bending vibrations in W-O and O-W. Similarly, studies on the stretching vibration of W-O-W linkages have yielded similar results [23]. The pH of the solution governs the nature of the types of present, and tungsten ions have a strong tendency to agglomerate in an acidic medium to produce a diverse spectrum of polyanions. Other studies have indicated that the broadband appearing in the 600–800 cm^−1^ range has been attributed to the O–W–O stretching modes present in the crystal structure of WO_3_ [24].

### 4.2. Antibiotic Susceptibility

In the past few years, antimicrobial resistance in mastitis pathogens has received attention due to the burden it imposes on lactating cattle in terms of drug toxicity and increased costs of therapeutics. Some studies on antimicrobial resistance were in line with the findings of the current study, but several other studies reported results in contradiction to that of the current study. The difference might be attributed to various factors, such as previous exposure to antibiotics, hygiene, consultation with a veterinarian, implementation of a mastitis control program, or overall animal health. This study contrasts with the findings of Singh et al. [25], who reported the high efficacy of ciprofloxacin against *E. coli* and *S. aureus*. As per the findings of the previous study [26], *E. coli* was found to be sensitive to erythromycin, while in another study it showed resistance to the same antibiotic [27]. In the case of *S. aureus*, other studies reported that gentamicin and enrofloxacin had the highest sensitivity, a finding that contradicts the results of current study [28]. In our study, amikacin, imipenem, and erythromycin exhibited the highest efficacy, whereas ampicillin was found to have the least, which is in accordance with the findings of Verma et al. [29]. The study conducted by León et al. [30] reported penicillin to be highly effective against *S. aureus*, which is in line with the findings of the current study. The utilization of antibiotics for therapeutic and nutritional purposes has resulted in a significant increase in the prevalence of pathogenic bacteria that exhibit resistance to many currently available antimicrobial agents.

### 4.3. Resistance Modulation by Nanoparticle Coupled Antibiotics

In the current study, nanoparticle-coupled antibiotics showed the highest MIC against *E. coli* and *S. aureus*. Like our findings, other studies on WO_3_-X nanodots have also shown significant bactericidal activity attributed to their membrane stress and photocatalytic properties, resulting in a significantly enhanced antibacterial activity [14]. Similarly, Liu et al. [31] showed that increasing the concentration of WS_2_ and prolonging the incubation time resulted in the eradication of both the *E. coli* and *S. aureus*. Previous reports have demonstrated that the antibacterial properties of WO_3_-X nanodots can be attributed to membrane stress and their photocatalytic properties [32]. A study conducted by Duan et al. [15], showed the remarkable antibacterial efficacy of tungsten oxide nanoparticles by a one-pot synthetic approach against Gram-negative *E. coli* and Gram-positive *S. aureus*. The antibacterial properties of tungsten nanoparticles were also investigated by Syed et al. [33] against *E. coli* and *S. aureus*, resulting in the inhibition of bacterial growth. In a study conducted by Ghasempour et al. [34], it was shown that tungsten oxide nanodots with diameters of 50–90 nm had antibacterial activity against *E. coli* bacteria when exposed to visible light irradiation. Moreover, it has been observed in other experimental findings that nanoscale metal oxides display antimicrobial activity depending on their exposure time to microbial cells, particle size, agglomeration process, and degree of degradation [35].

## 5. Conclusions

This study concluded diversified trends in drug resistance in individual *S. aureus*, *E. coli*, and mixed-culture bacteria. Tungsten oxide (WO_3_), on the other hand, exhibited substantial antibacterial properties against both individual bacterial strains and mixed-culture bacteria. Notably, as compared to non-coupled nanoparticles, WO_3_ nanoparticles coupled with antibiotics demonstrated the highest level of antibacterial potential. These nanoparticles exhibited pronounced antibacterial effects during the initial stages of incubation and have shown promise for application in the mitigation of bacterial resistance. However, for the purpose of establishing standardized benchmarks encompassing safety, effectiveness, and stability parameters, it is advisable to undertake further in vivo investigations and field trials.

## Figures and Tables

**Table 1:** Antibiotic susceptibility profile of *E. coli* and *S. aureus* against different antibiotics.

Antibiotic	*E. coli*			*S. aureus*		
	R (%)	I (%)	S (%)	R (%)	I (%)	S (%)
Imipenem	40	20	40	10	30	60
Amikacin	30	10	60	20	20	60
Oxytetracycline	30	20	50	40	20	40
Ampicillin	50	20	30	30	40	30
Gentamicin	40	10	50	30	20	50
Erythromycin	20	20	60	30	10	60
Ciprofloxacin	30	10	60	40	20	40
Penicillin	60	20	20	50	10	40

R = resistant, I = intermediate, S = sensitive.

**Table 2:** Comparisons of minimum inhibitory concentrations of nanoparticle-coupled antibiotics against *E. coli*, *S. aureus*, and their mixed culture.

Bacterial Culture Type	Preparation	Minimum Inhibitory Concentration (µg/mL) at Different Incubation Periods			
		4 h	20 h	24 h	28 h
*E. coli* alone	WA	520.83 ± 180.42 ^a^	65.10 ± 22.55 ^a^	52.08 ± 22.55 ^a^	39.06 ± 0.00 ^a^
	WO	312.33 ± 0.29 ^a^	65.10 ± 22.55 ^a^	39.06 ± 0.00 ^a^	32.55 ± 11.28 ^a^
	W	1458.33 ± 954.70 ^a^	260.42 ± 90.21 ^b^	130.21 ± 45.10 ^b^	78.12 ± 0.00 ^a^
*S. aureus* alone	WA	416.67 ± 180.42 ^a^	65.10 ± 22.55 ^a^	52.08 ± 22.55 ^ab^	26.04 ± 11.28 ^a^
	WO	312.33 ± 0.29 ^a^	52.08 ± 22.55 ^a^	26.04 ± 11.28 ^b^	19.53 ± 0.00 ^a^
	W	833.33 ± 360.84 ^a^	156.25 ± 0.00 ^b^	104.17 ± 45.10 ^a^	65.10 ± 22.55 ^b^
Mixed (*E. coli* + *S. aureus*)	WA	520.83 ± 180.42 ^a^	91.15 ± 59.67 ^a^	52.08 ± 22.55 ^a^	26.04 ± 11.28 ^a^
	WO	520.83 ± 180.42 ^a^	104.17 ± 45.10 ^a^	65.10 ± 22.55 ^a^	39.06 ± 0.00 ^a^
	W	2083.33 ± 721.69 ^b^	625.00 ± 0.00 ^b^	312.50 ± 270.63 ^a^	156.25 ± 0.00 ^b^

Different alphabetic (a, b) superscripts within the column of incubation for different preparations against each bacterial culture type indicate significant differences (*p* < 0.05); WA = tungsten oxide nanoparticle-coupled ampicillin, WO = tungsten oxide nanoparticle-coupled oxytetracycline, W = tungsten oxide nanoparticles alone.

## Data Availability

Not applicable.

## Supplementary Materials

The following supporting information can be downloaded at: https://www.mdpi.com/article/10.3390/biomedicines11112988/s1, Table S1. Comparative zones of inhibition (mm) produced by bacteria against different antibiotics.
