# Supplementary material for: Resistance Modulation of Individual and Polymicrobial Culture of S. aureus and E. coli through Nanoparticle-Coupled Antibiotics
Source: Biomedicines. 2023 Nov 7;11(11):2988. doi: 10.3390/biomedicines11112988 (PMC10669891; doi:10.3390/biomedicines11112988)
Supplement: Supplementary file 1 [file biomedicines-11-02988-s001.zip › biomedicines-2670085-supplementary.pdf]

Table S1. Comparative zones of inhibition (mm) produced by bacteria against different antibiotics.

Antibiotic	*E. coli*	*S. aureus*	Mixed Culture (*E. coli* + *S. aureus*)
Imipenem	16.33 ± 1.528 [a]	21.00 ± 1.00 [ab]	23.0 ± 3.00 [b]
Amikacin	11.00 ± 1.00 [a]	15.66 ± 1.528 [b]	9.33 ± 1.52 [b]
Oxytetracycline	10.00 ± 1.00 [a]	12.67 ± 2.08 [ab]	8.33 ± 1.52 [b]
Ampicillin	14.33 ± 1.52 [a]	12.67 ± 2.08 [b]	20.33 ± 1.52 [b]
Gentamicin	22.67 ± 2.52 [a]	33.67 ± 16.4 [a]	10.33 ± 1.52 [b]
Erythromycin	5.66 ± 1.52 [a]	5.00 ± 1.00 [a]	0.00 ± 0.00 [b]
Ciprofloxacin	10.00 ± 2.00 [a]	17.00 ± 2.65 [b]	5.00 ± 1.00 [b]
Penicillin	17.67 ± 2.52 [a]	19.33 ± 4.04 [ab]	11.33 ± 1.52 [b]

Different superscripts within a row indicate a significant difference ($p < 0.05$).
